# Approaching the vulnerability of refugees: evaluation of cross-cultural psychiatric training of staff in mental health care and refugee reception in Sweden

**DOI:** 10.1186/1472-6920-14-207

**Published:** 2014-09-27

**Authors:** Sofie Bäärnhielm, Ann-Sofie Edlund, Michael Ioannou, Marie Dahlin

**Affiliations:** Transcultural Centre, Stockholm County Council & Department of Clinical Neuroscience, Karolinska Institutet, Stockholm, Sweden; Transcultural Centre, Stockholm County Council, S:t Göran’s Hospital, Floor 13, 112 81 Stockholm, Sweden; Sahlgrenska University Hospital, Göteborg, Sweden; Center for Psychiatry Research and Education, Stockholm County Council & Department of Clinical Neuroscience, Karolinska Institutet, Stockholm, Sweden

**Keywords:** Culture, Training, Mental health, Refugees

## Abstract

**Background:**

This study evaluates the outcomes of cross-cultural mental health training given to professionals in health care and refugee reception in Stockholm, Sweden.

**Methods:**

A mixed method approach, with quantitative data from questionnaires (n = 232) and ten qualitative focus group interviews, was used.

**Results:**

After training, the participants reported that the hindering effect of lack of knowledge on their work decreased significantly from 2.81 (SD1.22) before, to 2.29 (SD1.00) (p < 0.001). Focus group interviews contributed to an understanding of this. According to findings from focus group interviews, after training, the participants shifted from emphasising communication barriers towards empathy with refugees with mental ill-health.

**Conclusion:**

Training resulted in an increased experienced capacity among participants to understand the social vulnerability of newly-arrived refugees with mental distress. However, the lack of collaboration and the structural barriers between the different organisations were not affected.

**Electronic supplementary material:**

The online version of this article (doi:10.1186/1472-6920-14-207) contains supplementary material, which is available to authorized users.

## Background

Refugees are at a high risk of developing poor mental health. Depression and Posttraumatic stress disorder (PTSD) are common reactions to losses and severe trauma [1]. In the cross-cultural context, which is a prominent feature of refugee reception, identification of mental health problems and correct diagnostic procedures are challenged. Culture shapes experience and expression of symptoms, signs of mental disorders and evaluation of behaviour. In DSM-5, the authors stress the importance of differentiating a mental disorder from a culturally approved response to stress or loss and of appreciating culture-specific presentations of symptoms [2]. Somatic symptoms are the culturally acceptable way of presenting depression in many cultural traditions, and symptoms and symptom clusters presented in PTSD and the meaning attributed to traumatic events may also vary according to culture [2]. In cross-cultural encounters, signs may thus be misinterpreted. Recently-arrived immigrants and refugees are at particular risk of being misdiagnosed in mental health care [3, 4].

In many European countries, migrants, asylum seekers and undocumented migrants in particular, have limited access to health services and social services [5]. In order to identify those refugees in need of mental health care and social support, staff in the field of refugee reception need to be knowledgeable about the variations in expressions of distress. Refugees’ access to care is thus affected by professionals’ knowledge about cultural aspects of patients’ expressions and understanding of mental distress. Further, the degree and quality of collaboration between agencies such as refugee reception and mental health care may influence refugees’ access to appropriate care. Refugee reception refers to the initial resettlement process and period when the new host nation is responsible for assisting the refugee. In Sweden, employment agencies today hold the overall responsibility for planning refugees’ introduction to Swedish society. The local social services are a main collaborator in the resettlement process.

### The Swedish context

Mental ill-health is more common among immigrants than native-born Swedes [6, 7] and refugees are especially exposed to poor somatic health [8]. Sweden has received several waves of refugees from various conflict areas of the world. Today, 15.1% of the population is foreign-born [9]. The main country of origin is Finland and the second is Iraq. The major groups of asylum seekers applying for permits to stay in Sweden during 2012 were refugees from Syria, Afghanistan, Somalia, Eritrea and those who were stateless [10].

All public health care is financed and organised by counties in Sweden. Mental health care is provided by primary care or specialised psychiatric clinics. Newly-arrived refugees with permission to stay have full access to health care, while asylum seekers are only offered subsidised care which cannot be deferred. At the time of this study, undocumented migrants in Sweden were excluded from subsidised health care. The situation has now changed and undocumented migrants have the same right to care as asylum seekers.

Until recently, refugee reception in Sweden has been organised by social services in the local municipalities but, as an effort to enhance integration within the labour market, since the end of 2010, employment agencies have been assigned the core responsibility. Education about cross-cultural mental health is thus required in order to reach the new professional groups and to address new challenges with regard to collaboration between organisations.

### Education regarding migrants’ mental health across organisations

To detect, evaluate and treat mental illness among newly-arrived refugees it is pivotal that the professionals who meet refugees have sufficient knowledge. Professionals working in refugee reception have had few opportunities for formal education or training in cross-cultural psychiatry. Knowledge of how culture may influence expression of mental distress is of particular importance. Education about refugees’ mental health has so far been limited even for students and professionals within the health and mental health care system in Sweden [11]. Further, there is lack of evidence about which training efforts are effective [11].

The Transcultural Centre in Stockholm operates under the auspices of Stockholm County Council. The Centre organises training and consultation support for health professionals in the fields of cross-cultural psychiatry and care for refugees and asylum seekers. With the goal of improving knowledge about culture, mental health, migration and trauma among professionals working within health care and refugee reception, as well as to facilitate collaboration between involved organisations, a training project was conducted and directed at professionals across the relevant organisations.

During 2011–2012 employees from the social services, the employment agencies, primary care centres and psychiatric clinics in seven municipalities in the Stockholm area participated in the cross-cultural mental health training. The training was organised as courses conducted locally in the municipalities. These courses were set up in collaboration with the local refugee reception services (social services and employments agencies), mental health care (primary and psychiatric care) and representatives from two national patient associations (one for patients with schizophrenia, *Intresseföreningen för schizofreni, Stockholm district,* and one for patients with various mental illnesses, *Riksförbundet för social och mental hälsa*).

Each course was held locally within each municipality, in premises free of charge. Courses were organised as one full day and two half days of training during regular working hours, scheduled over a period of two to three weeks to enable exchange between training and working experiences. The course comprised lectures and case presentations. The precise content in each municipality was planned with the local collaborators and included lectures covering: Migration’s effects on somatic and mental health, Culture and mental health, Migration and mental health in primary care, Recovery and shared decision making, Working with families in multicultural environments, Unaccompanied refugee minors, Trauma, Evaluation of working capacity, Stigma, Empowerment and, in one of the courses, Working with interpreters. In this paper, we report results from an evaluation of the training project.

#### Study aims

The overall aim of this study was to evaluate the outcomes of locally organised cross-cultural mental health training for staff in refugee reception and health care.

The specific aims were to:

1. study change in perceived knowledge and perceived barriers to their ability to do a good job among course participants before and after the course
2. identify perceived barriers and success factors that affected feasibility of the training
3. explore participants’ perception of barriers and success factors in encountering mental illness among refugees before and after the course.

## Methods

### Evaluation - mixed method design

A mixed method design of quantitative data from questionnaires and qualitative focus group interviews was used in order to enable both evaluation of quantitative outcomes of training and exploration of perception and meaning given to training. Quantitative research tests objective theories by examining relationships among variables and qualitative research explores individuals’ experiences as well as the meanings ascribed to the experiences by the individuals themselves [12].

### Quantitative sub-study

#### Study population

**Figure 1:**
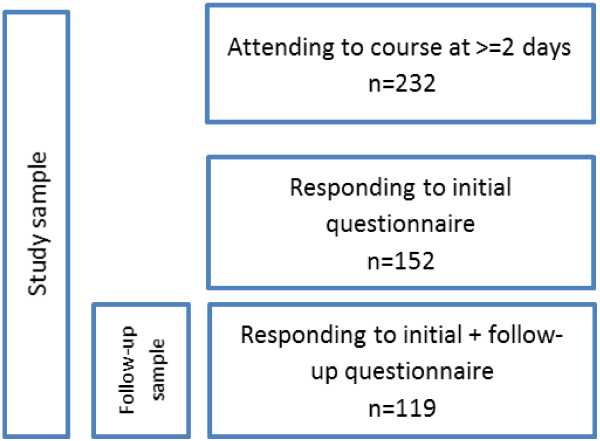
**Description of study population and sample.**

The study population consisted of 278 subjects who attended the local courses. The course participants were working in refugee reception within the local social services, in mental health care (primary care and psychiatric care) and in employment agencies. They had varying professional backgrounds, including social workers, Swedish language teachers, employment officers, nurses, psychologists and physicians. All participating organisations are publically funded. Each course was composed of three sessions, taking place on different dates. Only participants attending at least two sessions (N = 232) were included, see Figure 1. Ethical approval was obtained from the Regional Ethical Review Board in Stockholm, Dnr 2011/1227-31.

#### Data collection

All course participants were given a study identity number to enable individual follow-up, while ensuring anonymity. Identical questionnaires were distributed before and at the end of the final session. Data from included participants who responded to the questionnaire before participating in the course (T1) were used as a baseline, n = 152 (65.5%). The longitudinal sample, comprising those who had responded at both baseline and follow-up (n = 119, 51.3%), was used to evaluate change, see Figure 1.

#### Measures

The questionnaire was constructed for this study, since no validated instruments appropriate for the research questions were available. The questionnaires included questions on age, sex, and country of origin, whether participants themselves had a background as a refugee, had co-workers with a refugee background and whether they had contacts with refugees outside their profession. For evaluation of the course, perceived knowledge as well as perceived barriers to performing a good job was assessed before and after the course.

#### Perceived knowledge

Respondents rated their subjective knowledge with respect to 12 items of relevance to asylum seekers and refugees, the asylum seeking process in Sweden and health-related aspects. Knowledge in each item was rated on a 5-point Likert scale ranging from 1 = “completely insufficient for my work”, to 5 = “completely sufficient for my work”, see Additional file 1.

#### Barriers

Single item questions regarding predefined external barriers to performing a “good job”, such as lack of knowledge, lack of resources or poor cooperation within or between organisations, were rated on a five point Likert scale, between 1=”no barrier” and 5 = “major barrier”., see Additional file 1.

#### Statistical analyses

A factor analysis, (Principal component factoring with Varimax rotation, using the Kaiser criterion with Eigenvalues <1) was performed on the 12 perceived knowledge questions from the initial responders (n = 152). Mean scores for each factor before and after the course (n = 119) were computed. Comparisons were made for baseline mean scores between organisations by one-way ANOVA and for scores pre and post course by paired t-tests. All analyses were performed in PASW 20.0.

### Qualitative sub-study

#### Choice of qualitative method

A qualitative method was used in order to identify possible barriers and success-factors that affect the outcome and feasibility of this kind of cross-professional and cross-organisational education in cross-cultural psychiatry. This approach is particularly useful in situations where little research has been conducted. Qualitative data were collected by focus group interviews before and after the training. Focus group research is a useful tool for researching topics relating to group norms, the group meanings that underpin those norms and the group dynamic process whereby those meanings are constructed [13, 14] and are useful, on a contextual basis, for making culturally sensitive interpretations [15]. Focus groups are also useful in studying cultural values and workplace cultures, enabling access to communications that people use in day-to-day interactions, and highlighting subcultural and cultural values and norms [16].

#### Sampling and sample

A purposeful sampling was used [14] whereby focus group participants from psychiatric and primary care, social and employment services were recruited by the local training organisers. Most of the participants took part in both interviews. In some cases new participants were recruited for the follow-up interviews since some were not able to participate. This was done as focus group interviews reflect group experiences rather than individual experiences. Recruitment of participants from mental health care was hampered due to the limited possibility of being absent from clinical work. This was particularly the case for mental health professionals from in-patient settings. Some mental health professionals participated during their free time.

Five focus group interviews were conducted prior to training. In two local communities the organisers were not able to arrange focus group interviews before the courses. In total, 20 informants participated in the focus group interviews, 3 males and 17 females. Participants came from: psychiatric care (one), primary care (one), employment services (two) and social services (sixteen). The five follow-up interviews were conducted approximately one month after the last training session. Nineteen informants participated, 4 males and 15 females, from psychiatric care (one), primary care (one), employment services (two) and local services (fifteen).

#### Data collection

Focus group interviews were conducted shortly before and approximately one month after the completed training. The focus group interviews were conducted in the local municipalities and lasted approximately 1½ hours. Participation was voluntary and informed consent was given orally. The first author (SB) moderated most of the focus groups interviews together with a colleague observing group interaction. None of the interviewers participated in the training sessions, in order to support the participants to talk freely about the training. Semi-structured interview guides were used, see Additional file 2. In the pre-training interviews the participants were asked about their experiences of meeting health and mental health problems among newly-arrived refugees; their work in improving refugees’ mental health and difficulties in doing this; needs for improvements in their work; experiences of collaboration with other care providers in refugee reception and patient associations; expectations of training.

In the follow-up interviews participants were asked about their experiences of the training; how collaboration between the different agencies involved in refugee reception was affected by training; how their work with refugees’ health was affected by the training; their experiences of training outcome.

#### Data analysis of qualitative data

The interviews were digitally recorded and transcribed verbatim. Data from interviews before and after the training were analysed independently. For analysing data from focus group research, Bloor et al. [13] suggest a first step in processing transcribed texts from these groups, i.e. to index data in order to make them manageable for interpretation; the aim of indexing being to bring together all extracts of data that are pertinent to content.

Data were subsequently analysed using content analysis [17]. Basically, this consists of the interpretation of text through the systematic process of coding fragments of text (meaning unit), the classification of these codes in categories and the identification of patterns called themes [18, 19]. Collected data were initially organised according to content covered by interview questions into the three index areas: expectations/experiences of training; contextualisation of health; collaboration. After the first analysis of data the additional fourth index area of relations was identified. The initial analysis was performed by the second author (ASE) and then discussed with the first author (SB). For reliability check, results were finally discussed with the last author (MD), who had not participated in the focus group interviews or in the training activities. Analysis and coding were discussed until consensus was reached.

## Results

### Quantitative study

Of the 152 participants, 82.2% (125) were women, mean age was 44.2 (SD 11.7) and the average length of working experience within the field was 10.2 (SD 9.9) years. The majority of participants, 77% (117), worked within social services, 2.6% (4) at employment agencies, 5.3% (8) in primary care facilities and 15.1% (23) within psychiatric care. Almost one third, 30.9% (n = 47), had immigrated to Sweden, 15.1% (n = 23) had themselves a refugee background and 64.5% (n = 98) reported they had co-workers with a refugee background.

The factor analysis of the 12 questions regarding perceived knowledge revealed two factors, each containing six items; “Knowledge of support and care systems” (Cronbach’s α 0,88 ) and “Knowledge of health problems and treatment” (Cronbach’s α 0,90), see Table 1. Means at baseline ranged between 2.19 (0.63, psychiatry) and 2.50 (0.90, social services) for knowledge about support and care systems and between 2.79 (0.79, employment agency) and 3.10 (1.00, primary care) for knowledge about health problems and treatment, with no significant differences between organisations (data not shown).

**Table 1:** **Factor analysis of inventory on knowledge in areas pertaining to refugees and health, including 12 items (n = 152)**

Factor	I Knowledge of health problems and treatment	II Knowledge of support and care systems
Explained variance	35.3%	32.3%
Cronbach’s α	0.90	0.89
My knowledge…		
1. of rules and regulations for asylum seeking	.198	.822
2. of access to mental health care for asylum seekers	.388	.765
3. of available societal support systems to aid newly arrived refugees to settle	.248	.786
4. of available support to newly arrived refugees from voluntary organisations	.333	.807
5. of access to mental health care for newly arrived refugees with residence permit	.406	.692
6. of how migration may affect health	.679	.411
7. of how trauma may affect health	.787	.104
8. in detecting/recognising somatic ill-health among newly arrived refugees	.630	.445
9. in detecting/recognising mental ill-health among newly arrived refugees	.864	.296
10. of how to treat refugees with mental health problems or in crises in a good way	.715	.431
11. of treatment options for mental ill and psychologically traumatised newly arrived refugees	.757	.378
12. of support available from patient’s organisations, to those with mental ill-health	.631	.272

Principal Component Analysis with Varimax rotation, Kaiser Normalization.

The perceived knowledge about support and health care systems as well as about health problems and treatment for refugees, were both increased after the course (Table 2). The rated barrier effect of a lack of knowledge decreased from 2.81 (SD1.22) before, to 2.29 (SD1.00) after, the course (p < 0.001). There was no difference before and after the course in the perceived barriers towards the ability to do a good job from Poor collaboration within organisations, Poor collaboration between organisations, Lack of time, Lack of procedures or Lack of resources.

**Table 2:** **Ratings of perceived barriers and of perceived knowledge among staff from different organisations working with refugees, before and after training in transcultural psychiatry and migration, n = 119**

		Before training	After training		
		Mean (SD)	Mean (SD)	t	P
**Perceived knowledge**	Knowledge of health problems and treatment	2.43 (0.87)	3.32 (0.93)	-9.261	0.000
	Knowledge of support and care systems	2.89 (0.85)	3.75 (0.88)	-8.853	0.000
**Barriers**	Lack of knowledge	2.81 (1.22)	2.29 (1.00)	4.008	0.000
	Lack of cooperation within the organization	2.81 (1.16)	2.81 (1.27)	0.000	1.000
	Lack of collaboration between actors	3.28 (1.03)	3.23 (1.11)	0.390	0.698
	Lack of time	2.90 (1.21)	3.04 (1.11)	-1.215	0.227
	Lack of procedures	3.04 (1.18)	2.94 (1.14)	0.756	0.452
	Lack of resources	3.08 (1.17)	3.15 (1.92)	-0.491	0.625

### Qualitative sub-study

Results are presented along the four index areas of: expectations of/experiences from training, contextualisation of health, collaboration and relations, as summarised in Table 3. The quotes presented are chosen to reflect typical comments of participants.

**Table 3:** **Themes according to index areas, before and after training, from focus group interviews**

Index areas	Expectations of/experience from training		Contextualised health		Collaboration		Relations	
	Before training	After training	Before training	After training	Before training	After training	Before training	After training
Themes	Increased knowledge	Varying degrees of gained knowledge	Identified ill-health/distress	Increased resources to newly-arrived refugees with disability	Refugee reception staff needs health care actors to meet regularly	Insufficient collaboration on refugee needs	Massive barriers to communication	
	Collaborate	Wish for deeper knowledge	Explanations to ill-health	Limited ability to encompass all practical aspects concerning an individual	No collaboration with patient associations	Perceived structural barriers for refugees	Individual improvement strategies	I meet people who are vulnerable
	Uncertainty	Poor standard of premises sometimes hampered pedagogy	Difficult to help	Complex difficulties disrupt refugees’ capacity to study	Good experience from collaboration	Wish for structured local collaboration between agents	Responsibility and powerlessness	Increased empathy and emotional engagement

#### Expectations and experiences of training

Some participants had no previous training in either psychiatry or refugee health. Participants’ expectations of training were to acquire more knowledge about migration, psychiatric diagnoses and health. Participants also wanted to gain a better understanding of how they could contribute to improving refugees’ mental health and expressed uncertainty about how they could do it. A woman working in refugee reception in social services expressed her expectations:

*“What I think I experience quite a lot is this fear of being able to ask questions [referring to refugees], as an official person. Is it the right question, is it the wrong question?”*

Another expectation was that training would contribute to improved collaboration between different care providers so that refugees in need of a complex support package would receive better help. There was a fear that too difficult concepts would be used on the course. A man working in social services said:

*“I hope there won’t be words that are very academic. I am dyslexic and I have problems with such… ..but that it will be easy to understand.”*

After the courses the experience of improved knowledge varied. Some reported improved knowledge and others not. In some cases large groups in premises of poor standard was considered to have had a negative effect on training. Shared views were that local training close to the workplace was a positive feature, that there was a need for more concrete discussions about specific cases and that colleagues should receive the same training. Emotional aspects of learning were emphasised. A woman working in social services talked about her experiences of learning:

*“It’s probably important actually to have your feeling, your experience, reinforced. ‘Cause you experience so much in the encounters with so many different people and who are suffering mentally. There is so much you have to process by yourself, and this was actually a way to get some of what you experience up to the surface”*

#### Contextualised health

Before training, participants described how they identified different forms of ill-health among refugees and gave different explanations to it. The participants also talked about difficulties in helping refugees. They related experiences of encountering refugees with mental ill-health, health problems, sleeping problems, addiction, family conflicts, problems with daily functioning, and of refugees not expressing their mental distress verbally. Mental ill-health was often described as being expressed in bodily terms. A woman from the employment services said:

*“We have mainly encountered mental.., we have not met so many with physical ailments, at least they haven’t emerged; it is mainly mental illness. And sometimes it may be obvious that some are talking about their distress, in other cases it is more diffuse - you may sense that something is not very good.”*

Participants spoke about their own problems in helping refugees. This included shortcomings with regard to lack of knowledge, structural barriers, and of refugees receiving the wrong support. The woman from the employment services said:

*“ I recognise this feeling that it is all up to me now; this person does not know, cannot - and cannot speak the language - and is in distress. So if I don’t take action and make these contacts, then no one else will, and this person will go without help. It is terribly frustrating, because at the same time I feel that I am not competent to do this; I am not a psychologist or a doctor or- ah – this is also all new to us”*

After the training, participants asked for more resources for refugees with disabilities related to mental illness and pointed to their own shortcomings with regards to taking all practical aspects into consideration when dealing with the mentally ill. Participants emphasised the importance of social support and rehabilitation of refugees, especially for the mentally ill. They stressed the need for Swedish language education adapted for refugees with concentration difficulties.

In particular, participants appreciated the parts of the course where former patients had talked about their own experiences of mental illness. They also appreciated case discussions and practical examples of good care. A woman working in social services said:

*..it is always a good thing to hear how others handle or have handled these things, these aspects. I also think it is valuable to bring up and discuss successful examples, although the context may differ. ”*

#### Collaboration

Poor collaboration between the different agencies involved in refugee reception and mental health care was a common theme both before and after training. Before training, professionals from refugee reception expressed requests for mental health care that responded to the refugees’ needs. Refugee reception had no history of collaboration with patient associations. All participants expressed that they had little knowledge about the other care providers and how they worked. In several municipalities, the participants from refugee reception reported collaboration problems with healthcare; both psychiatric and primary care. Collaboration was described as satisfactory in one municipality. A participant from social services spoke about local collaborations:

*“Well, when this new assignment was initiated, there were collaborative working groups, to make local agreements between the social services and the employment agencies and so on… but there were no representatives from the health care organizations present in those”*

Collaboration was mostly dependent on individuals, but in the municipalities where it worked well, collaboration was anchored in the organisational structure. Many diverse care providers were involved in issues related to refugees and mental health. A woman from social services said

*“ ..so if I were to draw a network map of the all the people who need to be in touch with each other, that would make quite a few”*

After the training, participants described inadequate collaboration around newly-arrived refugees as well as experiences of structural barriers. Participants pointed to the ways in which problems with collaboration between organisations affected the health situation of newly-arrived refugees. They stressed the need for clear structures and local forums for collaboration. Further, participants emphasised the importance of understanding how other care providers and organisations worked and of having knowledge about the framework of their assignments.

A man working in social services expressed the importance of staff in refugee reception being informed of the treatment traumatised refugees receive from mental health care, in order to support and guide the refugees:

*“These so-called experts - who work with [traumatised refugees] on a daily basis – they could explain; and I think that would benefit all of us who meet these individuals existing in a no-man’s-land”*

#### Relations

Relations with refugees was a core area discussed before and after the courses. Before training, the participants talked about massive communication problems. Their strategies of overcoming communication problems were developed on an individual basis. Communication problems included difficulties in understanding refugees’ cultural backgrounds, other languages, and the use of interpreters in encounters. Participants described experiences of not being trusted by refugees, how stigma related to mental ill-health affected communication, and a desire for shared formulated strategies for overcoming the communication barriers. Participants reported carrying a heavy responsibility for refugees’ health situation, combined with feelings of powerlessness. A woman in social services said:

*“..well of course, you often do feel powerless. That’s natural; those are the things you struggle with each and every day”*

After the training, the participants described their work as involving encounters with refugees in a state of profound vulnerability and that the training had contributed to an increased understanding of the refugees’ situation and an increased emotional engagement. Participants’ discussions about how they related to refugees had shifted from identifying communication barriers towards emphasising the enduring and exposed social situations of refugees and other immigrant groups, and how the vulnerability of refugees affected them as professionals. This was a concern for both those who experienced that they had learnt plenty from training and for those for whom training mainly had meant a confirmation of knowledge.

Participants reported their own reactions in encounters with traumatised refugees. A man working in social services said:

*“..when there is someone with PTSD, I think a lot about my own background. I am from Bosnia and I have experienced war and known many fellow countrymen who have lived through horrible things and then*…”

For some participants the training had not involved acquiring much new knowledge but they still reported that the training had affected and improved their work. A woman working in social services said:

*“Well, although I just said there was nothing really new, it still confirmed what you have heard and you bring it with you to all further encounters. That is, one may be more receptive to things one may hear and notice more… like deviations… consider it more… yeah absolutely..”.*

Thus, training resulted in new perspectives on participants’ relations with refugees. Their capacity for approaching the social vulnerability of newly-arrived refugees with mental distress had increased. Participants’ focus shifted from emphasising communication barriers towards empathy with refugees in distress.

## Discussion

In this naturalistic study we evaluated outcomes of cross-cultural mental health training among professionals working in refugee reception and mental health care. The study was conducted in seven municipalities within the Stockholm area. Participants were a heterogeneous group regarding professional background and education.

The study was performed in a contextual situation of organisational changes. There had recently been a major re-organisation of refugee reception where much responsibility had been transferred from the local social services to the employment services. Further, the countries of origin for groups of refugees were changing. Refugees from the war in Syria had become the new and major group. Refugee reception in Sweden has been characterised by these types of change over the past decades.

### On method, limits and strengths

In this naturalistic study, we report pre- and post-effects on participants attitudes and perceived learning (Kirkpatrick level 1–2) [20]. This is a first step in a field where little research has been done. The use of identical questionnaires before and after the training enabled a comparison of perceived knowledge change beyond the mere rating of perceived gain of knowledge that a single post invention assessment would yield. We also think that the design, with training sessions held on three different occasions, thus facilitating a break between training and work, strengthens validity and relevance of findings. A further strength was the mixed method design, with a combination of questionnaires and focus group interviews to gain deeper understanding of the findings from the questionnaires.

Possible long-term effects of the training program, on practices (Kirkpatrick level 3) and recipient outcomes (Kirkpatrick level 4), would have been valuable to assess [20]. However, within this complex field, comprising different ways of organizing refugee reception, several different care providers and a high degree of system change, higher level effects of a training program are not likely to be detectable in a controlled manner. A limitation was that mental health professionals had difficulties participating. A strength was that the focus group interviewer did not participate in the training activities. However, the fact that the interviewer is a psychiatrist might have contributed to the participants shedding emphasis on mental health care.

### On results

The quantitative analyses showed that the participants’ perceived knowledge had improved after participating in the cross-cultural mental health courses. The perceived hindering effect due to lack of knowledge decreased significantly from 2.81 (SD1.22) before, to 2.29 (SD1.00) after the course (p < 0.001). From the questionnaires, it was not possible to grasp the content of the participants’ learning. However, the focus group interviews revealed that, after the courses, participants experienced an increased capacity for approaching the social vulnerability of newly-arrived refugees with mental distress.

Through training, participants had acquired new perspectives on their relations with refugees. After training, participants’ discussions about relations shifted from emphasising communication barriers towards an increased empathy with the refugees. For some participants, training mainly meant acquiring new knowledge. Concerning refugees’ contextualised health, our findings indicated that the professionals were in a process of re-orientation. They were trying to understand and cope with ways of expressing mental distress which were often unfamiliar to them and were struggling with how to be able to guide refugees to adequate help. There is a link between empathy and knowledge. Kirmayer [21] discusses the clinical limits of empathy in situations of radical cultural otherness. He points out that when empathy reaches its limits the other may be experienced as alien and unknown, and that empathy depends on detailed knowledge of the other’s world. Rasoal [22] points to how relationships in health care with clients from different ethno-cultural groups can be understood in terms of the presence or absence of ethno-cultural empathy. The findings from this study suggest that improved feelings of empathy may contribute to experiences of knowledge and an improved capacity for approaching the vulnerable situation of newly-arrived refugees and asylum seekers with mental distress.

The training was not enough to overcome the lack of collaboration between different organisations encountering newly-arrived refugees and asylum seekers. All participants underscored the importance of organisational structures for collaboration. Personal contacts and knowledge about the other organisation facilitated collaboration but was not enough.

### On development of cross-cultural training

Results from this study point to the importance of including educational components that support emotional aspects of learning in this kind of cross-cultural training. Such emotional aspects may include patient participation, personal narratives, case presentations and time for reflection. The need for cross- cultural mental training of professionals combined with a lack of studies of evaluation makes evaluation of training and various pedagogical aspects of cross-cultural training important. There is an additional need for research on teaching and learning methods and evaluating education and training in relation to service-user experiences and outcomes [23].

## Conclusions

After a cross-cultural mental health training intervention for staff in refugee reception and mental health care, we found a significant decrease in the hindering effect of lack of knowledge and that the participants experienced an increased capacity for approaching the social vulnerability of newly-arrived refugees with mental distress. Through training, participants had attained new perspectives on their relations with refugees. For some participants, but not all, training additionally gave new knowledge. With regard to relations, participants’ focus shifted from emphasising communication barriers towards empathy with the refugees.

## Electronic supplementary material

Additional file 1: **Translations from Swedish of Inventories of Knowledge and Barriers from the questionnaire.** (DOC 70 KB)

Additional file 2: **Semi structured interview guides.** (DOC 26 KB)
