# Supplementary material for: Approaching the vulnerability of refugees: evaluation of cross-cultural psychiatric training of staff in mental health care and refugee reception in Sweden
Source: BMC Med Educ. 2014 Sep 27;14:207. doi: 10.1186/1472-6920-14-207 (PMC4189165; doi:10.1186/1472-6920-14-207)
Supplement: Supplementary file 1 — Additional file 1: Translations from Swedish of Inventories of Knowledge and Barriers from the questionnaire. (DOC 70 KB) [file 12909_2014_1032_MOESM1_ESM.doc]

# Additional file 1.

# Translations from Swedish of Inventories of Knowledge and Barriers from the questionnaire

## Knowledge

Please rate your current knowledge within areas of relevance to this training.

| My knowledge.. | **..**is completely insufficient for  my work | | | **..** is completely  sufficient for  my work | | |
| --- | --- | --- | --- | --- | --- | --- |
| 1. of rules and regulations for asylum seeking |  |  |  | |  |  |
| 2. of access to mental health care for asylum seekers |  |  |  | |  |  |
| 3. of available societal support systems to aid newly-arrived refugees to settle |  |  |  | |  |  |
| 4. of available support to newly-arrived refugees from voluntary organisations |  |  |  | |  |  |
| 5. of access to mental health care for newly arrived refugees with residence permit |  |  |  | |  |  |
| 6. of how migration may affect health |  |  |  | |  |  |
| 7. of how trauma may affect health |  |  |  | |  |  |
| 8. in detecting/recognising somatic ill-health among newly-arrived refugees |  |  |  | |  |  |
| 9. in detecting/recognising mental ill-health among newly-arrived refugees |  |  |  | |  |  |
| 10. of how to treat refugees with mental health problems or in crises in a good way |  |  |  | |  |  |
| 11. of treatment options for mentally ill and psychologically traumatised newly-arrived refugees |  |  |  | |  |  |
| 12. of support available from patients’ organisations, to those with mental ill-health |  |  |  | |  |  |

## Barriers

Which of these act as barriers for you to do a “good job”

|  | **No  barrier** | | | **Major  barrier** | | |
| --- | --- | --- | --- | --- | --- | --- |
| Own lack of knowledge |  |  |  | |  |  |
| Poor collaboration within my organisation |  |  |  | |  |  |
| Poor collaboration between organisations |  |  |  | |  |  |
| Lack of time |  |  |  | |  |  |
| Lack of procedures |  |  |  | |  |  |
| Lack of resources |  |  |  | |  |  |
| Other, please describe below |  |  |  | |  |  |
