# Supplementary material for: Approaching the vulnerability of refugees: evaluation of cross-cultural psychiatric training of staff in mental health care and refugee reception in Sweden
Source: BMC Med Educ. 2014 Sep 27;14:207. doi: 10.1186/1472-6920-14-207 (PMC4189165; doi:10.1186/1472-6920-14-207)
Supplement: Supplementary file 2 — Additional file 2: Semi structured interview guides. (DOC 26 KB) [file 12909_2014_1032_MOESM2_ESM.doc]

# Additional file 2

# Semi structured interview guides

## Before training focus groups

Please tell me about your experiences of meeting health problems and mental ill health among newly-arrived refugees.

- What do you do to improve mental ill health among the refugees?
- What challenges do you experience?
- What needs to be improved in your work?

Please tell me about your experiences of collaborating with other organisations encountering newly arrived refugees.

- What difficulties do you experience?
- How does collaboration need to be improved?
- What are your experiences of collaboration with patients’ organisations?
- What local resources do you have in your community?

Please tell me about what you expect from the coming training.

**After training focus groups**

Please tell me about your experiences of the training.

- How has the training affected your organisation’s collaboration with other organisations encountering newly-arrived refugees?
- How has the training affected your work with mental ill health among refugees?
